# From R-Loops to G-Quadruplexes: Emerging New Threats for the Replication Fork

**DOI:** 10.3390/ijms21041506

**Published:** 2020-02-22

**Authors:** Antonio Maffia, Cecilia Ranise, Simone Sabbioneda

**Affiliations:** Istituto di Genetica Molecolare “Luigi Luca Cavalli-Sforza”, CNR, 27100 Pavia, Italy; antonio.maffia@igm.cnr.it (A.M.); cecilia.ranise01@universitadipavia.it (C.R.)

**Keywords:** replication fork, genome stability, R-loop, g4 quadruplex, common fragile sites

## Abstract

Replicating the entire genome is one of the most complex tasks for all organisms. Research carried out in the last few years has provided us with a clearer picture on how cells preserve genomic information from the numerous insults that may endanger its stability. Different DNA repair pathways, coping with exogenous or endogenous threat, have been dissected at the molecular level. More recently, there has been an increasing interest towards intrinsic obstacles to genome replication, paving the way to a novel view on genomic stability. Indeed, in some cases, the movement of the replication fork can be hindered by the presence of stable DNA: RNA hybrids (R-loops), the folding of G-rich sequences into G-quadruplex structures (G4s) or repetitive elements present at Common Fragile Sites (CFS). Although differing in their nature and in the way they affect the replication fork, all of these obstacles are a source of replication stress. Replication stress is one of the main hallmarks of cancer and its prevention is becoming increasingly important as a target for future chemotherapeutics. Here we will try to summarize how these three obstacles are generated and how the cells handle replication stress upon their encounter. Finally, we will consider their role in cancer and their exploitation in current chemotherapeutic approaches.

## 1. Introduction

Replication of the genome requires the coordination of highly dynamic mechanisms. During this process, DNA helicases unwind the parental DNA while DNA polymerases synthesize the new daughter strands. The group of proteins involved in genome duplication forms the so-called replisome. Factors that assemble to perform and regulate DNA replication are part of the replication fork. Although DNA polymerases have a pivotal role in the synthesis of nascent DNA strands, numerous other factors finely regulate the dynamics of the fork. 

The organised replication of the two DNA strands has to occur while counteracting exogenous insults and coping with intrinsic genomic obstacles. Cells attempt to repair DNA damage before S phase, when it may severely hinder the activity of DNA polymerases and consequently affect genome stability. Specific repair pathways have been evolved to cope efficiently with exogenous or endogenous insults, to sense and repair damage and to assist the replication fork [1]. Historically, one of the first types of damage studied was the damage inflicted by UV light. These wavelengths can generate adducts that distort the DNA double helix, such as cyclobuthane pyrimidine dimers (CPDs) or 6-4 photoproducts (6-4 PPs). The nucleotide excision repair (NER) pathway handles these lesions by cleaving the strand containing them and then filling in the gap, finally restoring the original DNA filament. Reactive chemical agents can modify nucleotide bases and generate aberrant products such as alkylated bases. These are recognised and repaired by the base excision repair (BER). Mismatch repair (MMR) assists the replication fork and corrects base mispairings thus preventing point mutations and in turn increasing replication fidelity. The most dangerous type of damage is the double strand break (DSB), in which ruptures to both filaments cause the disruption of the double helix. Two different pathways can repair DSBs according to the phase of the cell cycle. If a sister chromatid is present, during or after S phase, cells use the DNA from sister chromatids in a homologous recombination (HR) pathway to repair the breaks leading to a high-fidelity replacement. However, if a sister chromatid is not available the cells shift towards the non-homologous end joining (NHEJ) pathway that remodels the free DNA ends and then seals the breaks. Despite saving cells from more severe outcomes, this pathway can be highly mutagenic and it is responsible for the generation of insertions, deletions and point mutations. All of these repair pathways can work outside of the S phase and try to prevent the presence of DNA lesions at the arrival of the replication fork. However, some lesions may escape repair and persist until the S phase. The presence of DNA damage during the S phase can impair the normal progression of the replication fork. A distorted DNA template or a physical roadblock on the nucleic acid could slow or stall replicative DNA polymerases, leading to the uncoupling of DNA synthesis from the unwinding of DNA by the helicases. These disconnected activities might generate the accumulation of single stranded DNA (ssDNA) filaments [2]. ssDNA may be a dangerous intermediate, because it is more prone to breakage, and for this reason it is rapidly covered by replication protein A (RPA). RPA-bound ssDNA activates a signalling cascade known as DNA damage response (DDR). Ultimately, players of this pathway preserve cells from DNA damage caused by replication stress [3].

The Ataxia Telangectasia and Rad3 related (ATR) kinase is a pivotal node of this response and it is activated by the ssDNA-RPA covered intermediate. In turn, ATR phosphorylates and activates downstream targets that allow cell cycle control, protection of the replication fork and DNA repair. In essence, this pathway safeguards DNA replication during S phase despite the encounter of different obstacles by the replication fork [4]. In fact, ATR is considered a master regulator of a variety of pathways that protect the replication fork from arresting during genome replication. Interstrand crosslinks (ICLs) are a type of lesion that may stop the progression of the replication fork. These are generated by reactive chemical species such as aldehydes or platinum derivatives used in clinic as chemotherapeutics. To preserve DNA replication, these lesions are untangled by the Fanconi Anemia (FA) pathway [5]. Crosslinks, as well as other roadblocks, are detected by FANCM that activates the FA core complex made of different subunits and ubiquitylates and activate the FANCD2-FANCI complex. The active FANCD2-I complex recruits both the scaffolding protein SLX4 and endonucleases, such as *MUS81*, SLX1 and the XPF complex. These nucleases incise the strand containing the crosslink and generate both a double strand break and a DNA adduct. The DSB is repaired by homologous recombination while the extruded ICL is bypassed by translesion synthesis polymerases (TLS). In conclusion, this pathway removes forks impediments and restores the replication fork activity after its slow down. Other types of DNA distorting lesions that are not repaired before S phase can block the progression of DNA polymerases. The steric hindrance of the distorted double helix cannot fit the active site of replicative polymerases and requires the employment of alternative polymerases. Y-family polymerases possess a wider catalytic site, can accommodate such a template and bypass the lesion in a pathway called translesion synthesis [6]. These alternative polymerases are recruited at the level of the damage through Proliferating Cell Nuclear Antigen (PCNA) ubiquitylation that controls a regulated switch between the replicative and the Y-family polymerases. Their presence at the replication fork permits bypass of lesions such as those deriving from UV light. Despite their remarkable ability to perform lesion bypass, their wider catalytic site lowers their mismatch recognition on undamaged templates and thus makes them highly mutagenic. For this reason, their presence at the replication fork is tightly regulated via PCNA mono-ubiquitylation on lysine 164 (K164). The modification of the replicative sliding clamp is activated upon replication stress by a Rad6/Rad18 axis. The TLS pathway is a branch of a more general DNA damage tolerance pathway (DDT), that acts to preserve replication from replication stress occurring during S phase [7]. In addition to PCNA monoubiquitylation, K63-linked ubiquitin molecules can branch from K164 forming a polyubiquitin chain. This signal activates an alternative branch of DDT, the template switch pathway. Instead of bypassing lesions through the mutagenic TLS pathway, this pathway uses recombination to overcome obstacles in a high-fidelity manner. 

Other more complex mechanisms allow forks to bypass lesions encountered during replication. Fork reversal is a highly regulated process that prevents formation of DSBs intermediates to overcome fork blocks [8]. In this process, newly synthesized complementary DNA filaments are annealed giving rise to a structure that resembles a Holliday junction. Numerous factors are employed to remodel the fork, reverse it and protect it from nucleolytic activities. Reversal of the fork requires the activity of translocases that respond to replication stress and physically reverse the newly synthesized strands. The best characterised in this pathway are SMARCAL1, ZRANB3, HLTF and SHPRH. SMARCAL1 can bind directly to RPA filaments formed by ssDNA exposure, while the other translocases stimulate PCNA poly-ubiquitylation and bind to the modified clamp. The current model speculates that different translocases may recognize different fork intermediates and stimulate reversal. The formation of RAD51-covered DNA filaments is a crucial step in fork reversal and, together with both BRCA1 and BRCA2, they protect reversed forks from degradation. Finally, forks are remodelled by DNA2 and WRN helicase or by the RECQ1 helicase with the resolution of the junction and restart of the fork replication. The majority of such mechanisms have been recently documented and while providing unprecedented clues on forks reversal, further studies will be necessary in the future to address many of the molecular details of the pathway.

This brief summary of the cellular response to DNA damage exemplifies the incredible progress that has given us a better understanding on how cells protect the genetic material. However, although cellular responses to exogenous insults have been thoroughly characterized, new and more subtle threats are emerging. These new challenges for the replication fork have been identified and characterized only in the last decade. Replication stress describes a global disruption of the replication program. While the slowing down and the stalling of the replication fork have been extensively studied in the case of DNA damage, it is becoming clear that the metabolism of the nucleic acids in itself is a source of replication stress. During replication, the forks encounter a variety of protein complexes that are acting onto DNA, such as the transcriptional machinery. In fact, transcription can occur also during S phase when actively transcribed loci still need to be efficiently duplicated. This poses an interesting conundrum for the cells that have to juggle between DNA replication and RNA transcription. Collisions of the two machineries are a constant threat and need to be resolved especially if the two activities are directionally converging. In such conditions, triplex structures of RNA: DNA hybrids, that are normally transient, can be stabilized leading to pathological outcomes. These hybrids are called R-loops and it is becoming clear that they are important determinants for genome stability during replication and can account for replication stress.

In addition to the multiplicity of enzymatic activities that occur on DNA, the secondary structure of the nucleic acid and its topological status could also lead to problems during its duplication. If these situations are not dealt swiftly, they can result in a block and the eventual collapse of the replication fork. G4 quadruplexes, stacked secondary structures formed by G rich sequences exemplify such troublesome to replicate regions that are extensively scattered across the entire genome. G4s have been linked to transcriptional regulation of genomic loci and have been found to map with origin of replication underlying their importance in the metabolism of DNA.

Finally, DNA replication relies on several redundant mechanisms that allow for its completion. Not all the replication origins are fired at the same time during S phase and a specific temporal program is set and executed where multiple origins are kept dormant as a safety mechanism. These origins activate only when a local perturbation of replication occurs and their firing compensates for problems to nearby origins. It is envisaged that a region of the genome where the replication fork has been blocked could be rescued by passive replication from a newly activated dormant origin. 

Regions of the genome, such as common fragile sites (CFS), which are characterized by a low density of replication origins, are prone to breakage and show the importance of dormant origins activation as a safety mechanism.

Paucity of origins, secondary structures, and collisions between complex protein machineries on the DNA are very old threats for DNA replication that we have just started to investigate.

In this review, we attempt to present the different responses to replication stress arising from the inherent nature of the genomic sequence or from DNA metabolism, as well as the most relevant mechanisms to counteract them, ranging from R-loops to G4 quadruplexes and common fragile sites. In addition, we will address the importance of these pathways in cancer. The crucial role played by DNA damage in the rise of oncogenic phenotypes has been documented in depth, however how replication stress may trigger oncogenic signalling *per se* and how this affects tumour progression is still under scrutiny [9]. For these reasons it becomes crucial to dissect those pathways that control DNA replication dynamics ultimately providing new promising lines of therapeutics to treat cancer.

## 2. R-Loops

Threats to genomic stability not only come from exogenous events but also from the dynamic processes occurring along the double helix. One of such processes is RNA transcription. RNA polymerases, although they transcribe from DNA, can pose a serious problem to the replication fork. This issue is manifested especially in the genomic loci where both the replication fork and the transcriptional machinery are present concurrently on the same DNA template. This occurrence may lead to collisions between the two machineries and hinder both processes [10]. Many possible outcomes can be envisaged in these conditions, one of them being the slowing down of both replication and transcription that may stabilise transient DNA: RNA intermediates. These hybrid molecules, formed by the nascent transcript, template DNA and complementary DNA are called R-loops. Their name was derived from a similarity to another triple helical structure, the D-loop, which is formed during homologous recombination. The presence of R-loops has been detected across different species, ranging from bacteria to mammals, and they are now recognised as important players in both physiology and disease [11,12]. Their characterisation has required many efforts in the last few years in order to determine their presence along the genome in vivo, the mechanisms they regulate, and the pathways required to resolve the triple helix while preserving genome stability.

Different methodologies have been developed to sequence and precisely map R-loops along the genome, with the different experimental approaches leading to some discrepancies between results and spurring a debate on their location and their physiological role [13]. Some of these methods are based on a ChIP-seq approach and take advantage of pulling down either inactivated full length RNase H1, which degrades R-loops, or its hybrid binding domain [14,15,16]. However, most of the results were obtained by exploiting the S9.6 antibody that it is supposed to recognise DNA: RNA hybrids [17]. Thanks to its affinity, it is used to immunoprecipitate R-loops in a DNA: RNA immunoprecipitation protocol (DRIP) followed by sequencing (DRIP-seq) in different organisms [15,18,19,20]. However, the cross-reactivity of the S9.6 antibody towards secondary RNA structures or double stranded RNAs (dsRNAs) can affect its use. This issue has been mitigated by employing strict controls and by developing numerous technical improvements making DRIP based approaches the preferential technique to map R-loops with high resolution [11].

Genome-wide mapping of R-loops has allowed correlating their position with functional sequences in the genome. It revealed their presence at regulatory sequences of highly transcribed genes such as promoters or transcription termination sites [20,21,22]. Although R-loops can, at promoters, either stimulate or repress transcription, when formed at the 3′ end of gene loci they ensure an efficient and regulated termination of transcription [23,24]. The precise mechanisms by which R-loops influence gene expression are yet to be clarified but, undoubtedly, diverse lines of evidence have demonstrated their role in physiological processes.

### 2.1. R-Loops and Replication

Finely regulated R-loops arise to control gene expression but the unscheduled presence of this form of non-B DNA can become a source of replication stress if left unresolved. Dedicated pathways are present in the cells to control the formation and localisation of R-loops. By using different activities, a variety of enzymes are able to resolve the R-loop structure. All types of RNases H, H1 and H2, can degrade DNA: RNA hybrids with the former having an exquisite affinity for R-loops. In fact, RNase H1 over expression has been shown to counteract replication stress induced by R-loops [25,26]. Another strategy to prevent genome instability caused by R-loops, is an efficient and timely unwinding of the DNA: RNA hybrid. Numerous ATP dependent helicases, working on this substrate, have been characterised. The most relevant in humans are senataxin (SETX), FANCM, AQR, DDX19, DDX23, DDX1, DDX5, DHX9 and BLM [27,28,29,30,31,32,33,34,35,36]. However, in some cases they have been characterised mostly in vitro, whereas their role in unwinding R-loops in vivo is yet to be defined. The need for multiple helicases in their resolution may reflect the different roles that R-loops exert during transcription, with different partners working at promoters or terminators. It is plausible that some helicases may act either as part of the transcriptional complex or the replicative one. Another speculation is that their activity may be timely regulated in a concerted manner during different phases of transcripts elongation. Of interest, it was recently shown that mutant alleles of the transcription elongation factor TFIIS cause R-loops accumulation together with multiple defects in the dynamics of RNA polymerase II along transcribed regions [37]. This evidence further strengthens the interplay between factors of the transcription machinery and R-loops accumulation.

**Figure 1:**
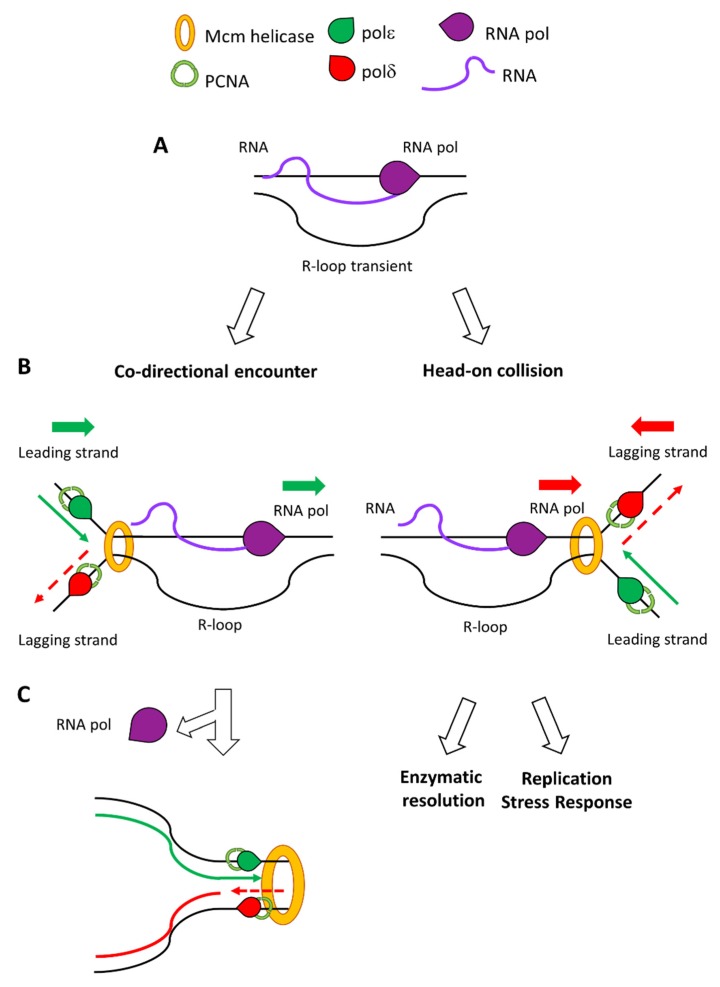
The orientation of the replication/transcription machineries determines the stabilisation of the R-loop and genome stability. (**A**) R-loops are formed by the triple helical interaction between template DNA, the nascent RNA transcript and the complementary DNA strand (**B**) When the RNA polymerase and the replication fork travel in the same direction, on the leading strand, the R-loop is displaced by DNA/RNA helicases associated with the fork (left panel). Differently, if the RNA polymerase moves towards the replication fork, on the lagging strand, the R-loop is more difficult to be resolved and may cause collision between the two machineries (right panel). (**C**) The respective direction of the travelling machineries determines consequences on genome stability. If the R-loop is displaced, then replication can continue unaffected (left panel). On the contrary, if the R-loop is stabilised by colliding transcription/replication it may require active resolution and cause replication stress (right panel).

Even though the cells employ multiple layers of control to prevent the presence of stable R-loops, some hybrids may escape these mechanisms and persist on the DNA template, threatening genome stability. In more detail, the presence of R-loops during S phase has been thought to occur due to transcription/replication collisions, a phenomenon that may have severe consequences for the genome [38]. The two complexes may clash in two different orientations: if they are moving on the same direction then they may cause a co-directional collision, while a head-on collision may occur if they are converging toward each other (Figure 1).

The two cases have different consequences in terms of DNA damage and fork stability. While both events are capable of stabilising R-loops, head-on collisions are believed to be more prone to cause replication forks stalling [39,40]. Differences between the dynamic properties of either complexes in the two different collision types have not been precisely elucidated yet, but it is believed that their respective orientation regulates the fate of the R-loop resulting in either its resolution or its stabilisation. At the moment, it is thought that in the case of head-on collisions the R-loop might not be accessible for resolution. Conversely, co-directional movement of the two complexes would allow the fork to reach the R-loop before it is stabilised by the pausing of RNA pol. This renders the hybrid available for resolution or displacement by the moving replication fork. 

**Figure 2:**
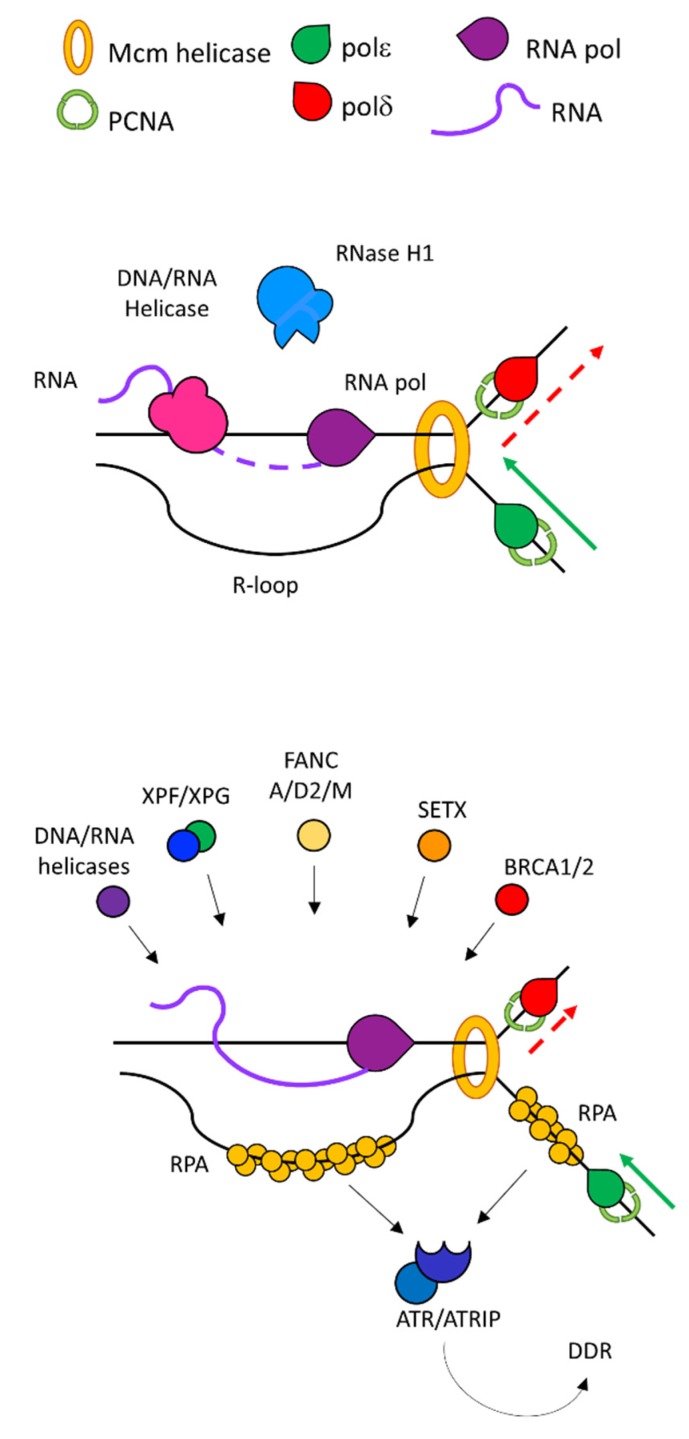
The resolution of R-loops determines the fate of head-on collisions. Different events occur on the two filaments according to R-loop resolution. The hybrid can be resolved by enzymatic cleavage by RNase H1/2 or unwinding by DNA: RNA helicases allowing fork restart (upper panel). If left unresolved, the R-loop triggers the replication stress response (lower panel). Different players are recruited to protect the fork, destabilize the R-loop, cleave or resolve the DNA: RNA hybrid. ssDNA filaments are covered by RPA that in turn recruits the ATR/ATRIP complex and initiates the DNA damage and replication stress response cascade.

A systematic explanation of how R-loops cause replication fork stalling is still ongoing. The current hypotheses speculates that different mechanisms may contribute to this phenomenon [41]. The roadblocks hypothesis considers that either the RNA pol or other factors pledged to solve the DNA: RNA hybrid may act as steric impediments to the moving fork. The unscheduled presence of stable R-loops causes further RNA pol pausing on the template, as demonstrated in vitro, and interferes with additional transcription of the target gene [31,42]. The presence of a paused RNA pol forms a roadblock for the movement of the incoming replication fork ending up with transcription and replication collisions [39,43]. Another intriguing hypothesis is that R-loops may induce epigenetic changes leading to chromatin condensation. This is substantiated by the presence of histones carrying post translational modifications characteristic of heterochromatin at the level of mapped R-loops loci [23,44]. A more compacted DNA may be more difficult to separate by the travelling fork and thus lead to replication fork stalling. This stalling may induce replication stress and DNA damage, namely breaks, unscheduled recombination and chromosomal rearrangements [11,45]. How breaks arise following R-loop stabilisation is yet to be explained in detail. The activity of nucleases could be responsible for the generation of breaks that may lead to unscheduled DNA degradation. A first hint towards this model was the demonstration that a deficiency in R-loops metabolism, due to the loss of AQR helicase and Senataxin (SETX), activates the transcription-coupled nucleotide excision repair (TC-NER) nucleases XPF and XPG [29]. Their activity accounts for the generation of DSBs, thus linking defective R-loop resolution with the generation of DNA damage (Figure 2).

Much progress has been done in characterising those factors that protect DNA or promote its repair when the forks are stalled by R-loops. Different lines of evidence have demonstrated that both BRCA1 and BRCA2 are involved in the cellular response to R-loop induced replication stress. Loss of BRCA2 leads to the accumulation of R-loops and the activation of the DDR pathway. Interestingly, this phenotype is reversed by the over expression of RNase H1 [16]. The inactivation of BRCA genes in cancer causes an increase of mutations at the level of gene bodies, regions that are characterised by higher negative supercoiled DNA. This topological phenotype is frequently associated with transcription stress caused by R-loops further strengthening the roles of BRCA1/2 in fork protection against stable R-loops [46]. Moreover, BRCA1 has been shown to recruit SETX to transcription termination sites that are prone to form R-loops [47]. At those loci, BRCA1 mutated cancers show accumulation of insertions or deletions [48]. All this evidence demonstrates how BRCA1 is necessary to prevent mutagenicity arising from DNA instability as a consequence of unresolved R-loops. 

The role of BRCA2 is more puzzling, especially considering its multiple activities in diverse aspects of the DNA damage response and the cell cycle. For instance, persistence of BRCA2 onto chromatin can be reversed by RNase H1, showing a link between its presence and DNA: RNA hybrids metabolism [16]. Compelling evidence has demonstrated that BRCA2 interacts with RNA pol II and promotes its dissociation from pausing sites, thus decreasing R-loop accumulation and DNA damage [49]. The RNA pol II associated factor 1 (PAF1) has a crucial role in this process and inactivation of BRCA2 decreases PAF1 recruitment to RNA pol II causing both R-loops accumulation and DNA damage. This is not the unique indication suggesting an interplay between BRCA2 and regulators of mRNA maturation. BRCA2 can interact with PCID2, a subunit of the TREX-2 complex, involved in mRNP metabolism and trafficking [16]. It is speculated that TREX-2 could mediate the recruitment of BRCA2 at naturally occurring R-loops during transcription. BRCA2 binding may help expose the branched structure formed by DNA: RNA hybrids making the R-loop more accessible for resolution, by either RNase H1 or various helicases (e.g., SETX).

Taken together this evidence suggests the involvement of the tumour suppressor BRCA2 with the transcription machinery allowing a correct mRNA biogenesis and, in turn preventing R-loops accumulation and DNA damage. In addition to its interplay with the mRNA biogenesis factors, BRCA2 can relieve stress from R-loops by protecting stalled replication forks from degradation by nucleases, such as Mre11 [50,51]. Despite its potential toxicity, degradation of the forks by Mre11 is one of the first steps in forks remodelling upon stalling. The cells use this pathway as a fork rescue mechanism and it requires a fine balance between BRCA2 and various nucleases [52]. Fork reversal is speculated to be an additional mechanism by which the cells relieve replication stress by R-loops. Head-on encounters of the replication fork with the transcribing RNA pol cause fork reversal with the generation of RAD51-covered DNA filaments [53]. RECQ5 and RECQ1 helicases may mediate fork reversal while its remodelling is performed by the *MUS81*/EME1 nucleases. RAD52 and Ligase 4 (LIG4) finally restore the replication fork in a process that requires active transcription. Upon R-loops accumulation and fork reversal, ATR is activated by *MUS81* and it mediates cell cycle arrest through the Chk1 kinase [54]. In turn, ATR controls *MUS81* to prevent uncontrolled forks cleavage, establishing a finely tuned control loop that oversees fork reversal. ATR is speculated to be activated also in the absence of fork reversal either by the recruitment to RPA covered ssDNA at the stalled replication fork or, in a less canonical way, at the level of the displaced ssDNA helix of the R-loop itself (Figure 2) [11]. Dissecting this pathway at the molecular level will add some fundamental insights on how the forks respond in a dynamic manner to replication stress by R-loops. 

Compelling evidence shows how different cellular repair systems might relieve stress caused by R-loops. The Fanconi anemia (FA) pathway was discovered as a mechanism that repairs intra-strand and inter-strand DNA crosslinks (ICLs) [28]. In addition, its activity has also been linked to repression of DNA damage induced by R-loops. A higher number of R-loops was detected by DRIP in cells defective for either FANCD2 or FANCA [55], two of the crucial components of the Fanconi anemia pathway. Furthermore, the FA pathway was also shown to protect forks from stalling as they encounter R-loops. The FANCM helicase prevents fork arrest with its translocase activity when R-loops accumulate following FANCA and FANCD2 depletion [55]. An interplay between BRCA2 and FA pathway has been documented. BRCA2 was shown to interact with activated FANCD2 in the absence of exogenous replication stress, postulating that such an interaction may also work in the case of R-loops induced DNA damage [56]. A more detailed study on the interplay between these pathways will be of fundamental importance to understand how these factors counteract R-loops dependent DNA damage. The current hypothesis suggests that BRCA2 may have a role in recognising R-loops and then activating the FA pathway to protect the replication fork. 

The presence of R-loops may partially explain the instability of a subset of regions of the genome [57]. R-loops have been identified at the level of fragile sites [44,58]. The concept that repeated sequences may form R-loops was already demonstrated both in vitro and in vivo by monitoring instability of repeats after RNase H1 or H2 knockdown [59,60]. The presence of R-loops at these sites is linked to the activity of the FA pathway. For example, DNA: RNA hybrids accumulate at FRA16D, when FANCD2 is absent, causing replication stress that is relieved by the overexpression of RNase H1 [61].

These are not the sole loci where R-loops may be responsible for genomic instability. It has been speculated that G-rich sequences in the non-template strand of the R-loop structure may form a G4 motif. These structures have been observed in vitro during active transcription and have a role in stabilising the R-loop itself [62]. Recently, an intriguing interplay between G-quadruplex and R-loops has been found in cancer cells. It was shown that stabilisation of the G4 motif in cancer cells causes a spread of R-loops downstream of transcribed loci [63]. This, in turn, triggers genomic instability at these regions affecting transcription. Expression of RNase H1 relieves this genomic instability. Thus, the combined presence of an unresolved G4 motif and a stable R-loop could underlie defects in transcription efficiency ultimately leading to DNA damage at the locus. In addition to this evidence, an overlap between R-loops and non-B DNA forms such as G-quadruplexes has been predicted by genome wide computational analysis of R-loops forming loci [64]. Interestingly the majority of these sequences co-localised at functional regions such as promoters, gene ends and enhancers. These novel findings provide an exciting new interplay between regulatory sequences that would be of enormous interest for future discoveries on both transcription regulation and genome stability. 

### 2.2. R-Loops and Cancer

Since R-loops have a direct role in the stability of replication forks and if they are not properly handled can cause DNA damage, their mis-regulation may boost oncogenic phenotypes. The interplay between R-loops and oncogenic signalling was demonstrated in the case of the estrogen receptor pathway in breast cancer [26]. Here, genes induced by estrogen accumulate R-loops and are subject to translocations. This supports the idea that stress induced by oncogenes activation may cause accumulation of R-loops that, in turn, increases DNA damage. This is not the only oncogenic pathway that causes an accumulation of R-loops. A global increase of transcription caused by HRAS overexpression leads to R-loops stabilization, resulting in replication stress and instability [25]. Thus, the presence of R-loops may help predicting the loci that undergo instability in an oncogenic background. Accumulation of R-loops may also occur by the loss of tumour suppressors such as BRCA1/2 [65,66]. In an opposite manner, accumulation of R-loops may trap these factors causing their functional depletion. For instance, the EWS-FLI1 protein, found in patients affected by Ewing sarcoma, is able to trap BRCA1 by blocking its physiological role in protecting replication forks [67]. Similarly, BRCA2 can be sequestered by R-loops impairing RNA pol II release from transcription pausing sites and exacerbating both R-loops accumulation and RNA pol II blocks [49]. Exploiting synthetic lethality with PARP inhibitors in cells that are BRCA1/2 deficient may be a promising strategy to target tumours that present a high level of R-loops [67]. Other strategies have been proposed to target DNA damage pathways that are activated in response to replication stress induced by R-loops. With this in mind, synovial sarcoma cells treated with an ATR inhibitor accumulated R-loops and DNA damage resulting in increased apoptosis [68]. Killing of tumour cells was even more pronounced when ATR inhibition was combined with agents that affect replication fork progression such as cisplatin or PARP inhibitors. This evidence clearly shows how targeting R-loops may be promising as either a secondary line or a combinatorial strategy of treatment for novel chemotherapeutic strategies. 

## 3. G-Quadruplex

The conformation of the DNA double helix affects its metabolism with no exception for replication. Alternative forms to the classical right-handed B-DNA have been recognised years ago [69]. These comprise a variety of structures such as cruciforms, triplexes, H-DNA, Z-DNA and G-quadruplexes. In most cases, these forms are due to the repetitive nature of genomic regions and are susceptible to phenomena of genetic instability. This intrinsic instability could underlie the development or progression of neurodevelopmental disorders and cancer [70]. Recently, G-quadruplexes are in the spotlight because of their increasing relevance in both physiological and pathological conditions [71].

**Figure 3:**
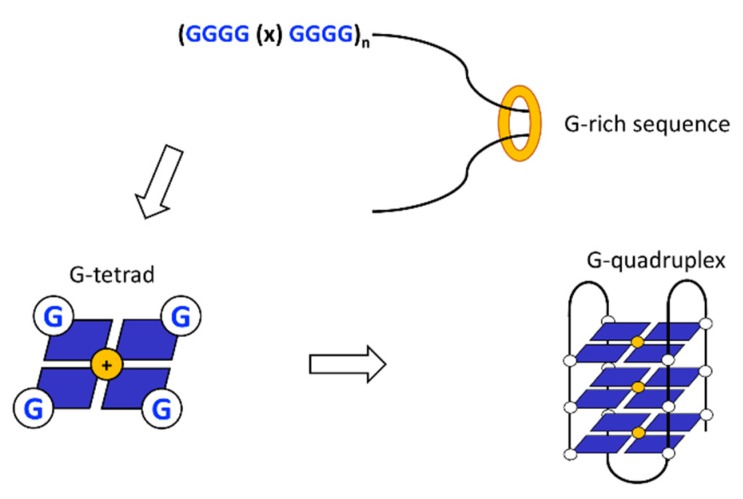
G-quadruplex DNA is formed by G-rich sequences. Guanines at G-rich sequences can interact to form higher order structures. If stabilised by the presence of monovalent cations, guanines can form a planar structure called G-tetrad or G-quartet by Hoogsteen hydrogen bonds. Multiple planar structures can stack onto each other to form bi-, tri-, tetramolecular G-quadruplexes.

These non-canonical DNA secondary structures form by the interaction of guanines in G-rich sequences where nucleotides interact via a Hoogsteen hydrogen bond stabilised by a cation. These interactions organise the nucleotides in a planar conformation called G quartet. Planar G quartets may stack by π–π interactions to form G-quadruplexes (G4s) (Figure 3). Different strands can participate in the G4 structure forming unimolecular, bimolecular or tetramolecular G4s with the former being the most common detected in vivo.

Early studies have demonstrated the formation of these structures of DNA at physiological salt conditions, paving the way to their molecular characterisation in the following years [72]. While being extensively studied in vitro, their presence and relevance in vivo has been documented only in the last decade. G4s were initially predicted in silico, then identified in mammalian cells by newly developed G4s specific ligands and only recently they have been mapped at genome wide level [73,74,75,76,77]. The functional relevance of these sequences has long been debated. Their high level of conservation, from yeast to mammals, suggests that such structures may have a role in regulatory regions of the genome. In line with this hypothesis, G4 motifs were first identified at telomeres, which are typically GC rich regions [78]. In addition, G4s were found within promoters of oncogenes [79,80], at replication origins [81] and CpG islands [82]. The importance of these sequences in numerous physiological processes is now becoming increasingly clear. Studies on the role of G4s can now rely on the development of novel G4 ligands that allow their detection in different cellular processes in vivo [83]. At present, the function of G-quadruplex DNA has been already defined in different regulatory pathways and they appear to play a role in the control of transcription and even the firing of origin of replication. While G4s exert an important physiological role they can become an obstacle to the replication fork and cause DNA damage [71].

### 3.1. Replication of G-Quadruplex DNA

**Figure 4:**
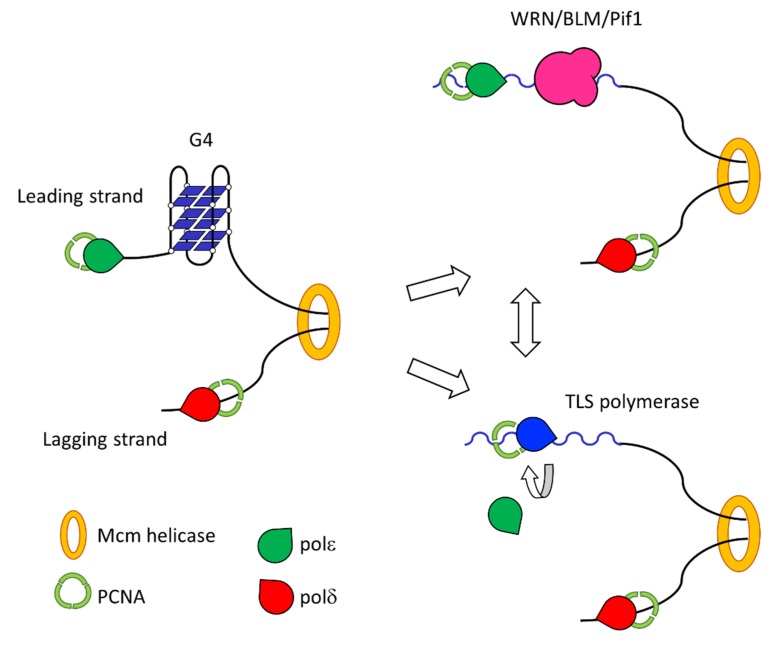
Resolution of G4 motifs after replication fork block. The presence of a G4 motif can impair the movement of the replication fork (left panel). This block can either be resolved by helicases (upper right panel) or by a switch between replicative and Y-family polymerases (lower right panel). The two mechanisms are not mutually exclusive: helicases may melt the G4 that TLS polymerases may then bypass.

DNA replication transiently exposes ssDNA, especially during lagging strand synthesis. This intermediate is more prone to fold into G4s and thus hinder movement of the replication fork. The inability of the replicative polymerases to move past G4s has been demonstrated in vitro [84,85]. In vivo evidence of such impediments came at first from deletion of the helicase FANCJ in *Caenorhabditis. elegans* [86,87]. Cells lacking FANCJ accumulated short deletions mapping near G4 rich regions, consistent with a block of the replication fork at these sequences. Together with FANCJ, numerous helicases have been implicated in the resolution of the tetraplex to prevent stalling of the replication fork. RecQ family helicases, in particular BLM and WRN, have been shown to be able to resolve G-quadruplexes [88,89]. In addition, the evolutionary conserved Pif1 helicase is able to suppress genomic instability caused by G4s accumulation [90] (Figure 4). Helicases can actively remodel DNA, unwinding the strands in an-ATP dependent or independent manner [91]. By sliding through the DNA filaments, these enzymes can melt secondary structures making the template DNA suitable for the incoming polymerases.

The relative contribution, in the unfolding of G4s, by each of the different helicases, is still puzzling to scientists in the field. It is widely accepted that they may have different affinities for different DNA structures generated when the replisome encounters structured DNA. Unwinding of structured DNA by helicases is not the sole strategy the cells employ to deal with the hindrance of G-quadruplex DNA. For instance, translesion synthesis has a role in replicating past G4 motifs. The slowing down of the replication fork in front of G-quadruplexes may be the reason for the recruitment of Y-family polymerases. The higher versatility of these polymerases in terms of bypassing distorted templates could be useful in replicating through G4s. Among the different alternative polymerases, Rev1 has a relevant role in this scenario. Rev1 is a deoxycytidyl transferase that can catalyse insertion of a dCMP molecule to the 3′ end of a primer in front of a guanine [92]. Efficient and timely bypass of structured DNA, as well as any distorted template, is required to preserve the chromatin status of the locus and its epigenetic marks [93]. Any delay in the process is thought to result in an impediment in recycling parental histones carrying epigenetic information, leading to the incorporation of new naïve histones devoid of these crucial modifications. In DT40 chicken cells the expression of the β-globulin locus is silenced in non-erythroid cells by deposition of repressive methylated histone H3 (H3K9me2) [94]. Rev1 deficient cells showed a change in the epigenetic marks at the globin locus with a loss of histone H3 methylation and an increase in histone H4 acetylation [95]. This change in epigenetic status of the region correlated with the presence of a G4 in the locus and the inability of the cells lacking Rev1 to replicate efficiently in that genomic region. 

Both the C-terminal domain of Rev1, involved in interactions with other TLS polymerases, and the catalytic domain were shown to be required to replicate past the G4 sequence. This implies that Rev1 may facilitate replication through structured DNA via interacting with alternative polymerases and bypassing the G rich sequence thanks to its deoxycytidyl transferase activity. This evidence was confirmed by a similar experimental model, where a single G4 motif is located on the leading strand upstream the transcription start site of the BU-1 gene in chicken cells [96]. The role of Rev1 in G4 instability may be explained by its ability to melt the G4 structure and prevent its refolding as demonstrated in vitro [97]. Taken together, Rev1 is required for destabilisation of the G4 motifs, in concerted action with FANCJ [98], and for bypassing guanine motifs through its deoxycytidyl transferase activity. These events would assist the fork in the immediate encounter with the G4 preventing a deleterious fork stalling. Further investigation on the role of TLS polymerases has focused on polymerase η, polymerase κ and polymerase ι [99]. Their role into G-quadruplex dynamics was assessed in vivo by the use of telomestatin, a G4 stabilising compound. Combination of telomestatin and silencing of any of the three polymerases demonstrated that only polη and polκ were fundamental for the survival of the cell. The decrease in cell viability, following knock down of the alternative polymerases, was related to an increase of DSBs generated by replication fork stalling. More recently, studies in vitro have further strengthened the hypothesis of a role of polη in replicating past G4 motifs [100]. Polη was shown to efficiently elongate primers in front of a stable G4 structure with a higher fidelity than the replicative polymerase polε. A more thorough investigation of the role of TLS in replicating past G4 motifs is necessary. However, it is becoming increasingly clear that replication stress generated by the encounter of quadruplex DNA is alleviated by the recruitment of alternative polymerases [101]. 

### 3.2. G-Quadruplex and Cancer

Different experimental approaches have led to the identification of G4 motifs with a functional role at numerous gene promoters [102,103,104]. As an example, it was recently demonstrated that a G-quadruplex structure inhibits methylation of CpG islands locally, by sequestering DNMT1 [82]. Such studies have leveraged the hypothesis that the presence of G4 DNA may provide cells with an additional mechanism of transcriptional regulation of nearby genes. The importance of transcriptional control by G4 structures is critical at promoters of oncogenes. It has long been known that both c-MYC and KRAS oncogenes have a G4 motif within their promoters [79,80]. In both cases, G-rich tracts fold into a G-quadruplex structure upstream of their respective promoter. These structures can be further stabilised by cationic porphyrins causing a reduction of transcription to the downstream gene. To corroborate this hypothesis, SRC was also shown to present a G4 motif in its promoter and stabilisation of the quadruplex structure, by use of small-molecule ligands, reduced the activation of the proto-oncogene [102]. These cases present a clear correlation between the presence of G4 motifs and oncogenes expression. G4s structures were identified also at the promoters of other oncogenes, namely c-KIT, BCL2 and VEGF [105,106,107]. Once an oncogenic pathway is activated, this activation boosts replication stress. When tumours accumulate replication stress the nucleotide pool is rapidly depleted [108]. Artificial depletion of nucleotide pools by hydroxyurea (HU), induces changes in the gene expression profile that resembles the transcriptional perturbations induced by the loss of helicases involved in G4 resolution [109]. Thus, induction of alternative gene expression profiles is linked to the presence of G4 motifs that may remain unresolved during cancer progression.

Resolution of the quadruplex structure is also fundamental to prevent genomic instability that could sustain a tumorigenic phenotype, as exemplified by the increase in DSBs after stalling of the replication fork in front of a G4 [102]. Data coming from either genome wide sequencing of G4s or ChIP-seq experiments identified copy number variations, in particular amplifications, at the level of G-quadruplexes as a result of chromosomal breaks [77,103,110]. Interestingly, many of the identified G4 containing loci mapped with oncogenes, tumour suppressors and copy number variations that are frequently found in cancer [77,111,112]. More strikingly, a higher number of G4 motifs were detected by immunohistochemistry in tumour tissues coming from patients affected by either stomach or liver cancer [113]. Nowadays we have a strong evidence of the role of G-quadruplex DNA as an important player in cancer progression. For this reason, the design of small molecules that bind the G4 structure is an appealing drug targeting strategy [71,114]. A primary issue with this approach is the improvement of selectivity both towards the quadruplex structure, as opposed to dsDNA, as well as towards specific types of G4s. Targeting an oncogenic G4 out of a variety of physiological structures is a major challenge. NMR and X-ray crystallography have provided useful hints about the specific conformations of defined G4s. Despite sharing a similar general structure, some G4s can have peculiar loops and grooves that may exploited for precise targeting. Multiple outcomes can be expected by targeting G4s with small molecules. The RHSP4 molecule kills cancer cells by targeting telomeric DNA and causing telomerase inhibition and DNA damage [102,115]. Small molecules may also be designed to target G4s at oncogenic promoters to downregulate downstream gene expression. With this in mind, MYC was targeted with an ellipticine derivative that causes downregulation of MYC expression in non-Hodgkin lymphoma [116]. Some molecules may also act across multiple pathways to exert their anti-tumoral activity. In this direction, EMICORON is a very promising compound showing a good efficacy in vivo towards colon cancer models [117]. The compound destabilizes telomeric DNA but also downregulates both BCL2 and MYC by binding their promoters [118,119]. This broader activity may explain its efficacy. The interplay between G4s and genome instability may also be exploited to cause cancer cells death. G4 binders may efficiently target tumours carrying mutations in the DNA damage response, such as BRCA1 and BRCA2. Synthetic lethality has been exploited in such genetic backgrounds by combining DNA damage sensitivity with stabilisation of G4s. The small molecule G4 stabilizer pyridostatin (PDS) has a higher efficacy when targeting homologous recombination (HR) deficient cancer cell lines. In fact, a BRCA2 -/- genetic background or depletion of either BRCA1 or RAD51 confers a higher sensitivity to PDS in HCT116, DLD1 and HEK293T cancer cell lines [120,121]. RHPS4 has a stronger activity towards BRCA2 deficient tumours by increasing DNA damage that cannot be repaired by cells [121]. Two novel compounds, quarfloxin and CX-5461, have now entered phase II and phase I clinical trials respectively. Both molecules have shown an exquisite anti-tumoral activity towards BRCA1/2 deficient tumours with no adverse effects [122,123]. Understanding how G4 motifs are processed and especially how they are replicated to avoid genomic instability, may provide additional strategies for combined chemotherapeutics. For instance, the combination of RHPS4 with PARP inhibitors substantially reduces colon cancer progression in mice and increases their survival at higher extent than the administration of single compounds [115]. With a similar mechanism, by inhibiting the helicase WRN, cancer cell lines are more sensitive to telomestatin [124]. It is now clear that G-quadruplex DNA not only has a role in relevant physiological pathways, such as development, but may also become an appealing target to find new strategies in drug design for cancer chemotherapy. 

## 4. Common Fragile Sites

As previously mentioned, the progression of the replication fork along the genome can be hindered by the inherent nature of the sequences it encounters. A clear example of this scenario is given by the replication of common fragile sites (CFSs). These genomic regions have received an increasing interest because they undergo gross chromosomal rearrangements in tumours. However, the correlation between their role in cancer and their mechanisms of replication is a recent discovery. Seminal cytogenetic studies showed that these regions were exquisitely sensitive to replication stress and treatment of the cells with aphidicolin, an inhibitor of DNA polymerase α, resulted in breaks in metaphase chromosomes [125]. The molecular characterisation of this sensitivity has unravelled different peculiarities of these loci that explain their fragility. Sequencing of breakage sites revealed that CFS are AT-rich regions, prone to form secondary DNA structures and non B-DNA [126,127,128]. The presence of non-canonical forms of the double helix influences progression of the replication fork and it is one of the causes of chronic replication stress at the level of these regions [129]. In addition, when forks are challenged during S phase, an ATR mediated pathway can prevent instability by controlling firing of late or dormant replication origins [130]. When the ATR-CHK1 axis is activated by replication stress, it inhibits global origin firing while it promotes local activation of nearby dormant origins [4]. This prevents spreading of defective replication globally but rescues stalled forks locally. However, fragile sites present a scarce density of origins thus preventing the use of such a rescue mechanism. Origins located at the FRA3B site fire less efficiently upon replication stress and cells that carry breaks at this site show less active origins [131,132]. Mapping of origin recognition complexes (ORC) binding sites along the human genome has revealed their paucity at CFS [133]. In addition, their scarcity correlates with mapped CFSs and regions carrying deletions in cancer. This data clearly underlie how replication through fragile sites is deprived by a possible rescue mechanism through dormant origins activation. This feature is exacerbated further by the length of CFSs, which requires the fork to travel long distances without having the possibility to be rescued. Mapping breaks at FRA3B and FRA16D revealed that these fragile sites lye within large genes: the 1.3 Mb FHIT gene and the 1.1 Mb WWOX gene [134,135,136]. Sequence composition and physical characteristics of these sites do not completely account for their propensity to break. Active transcription of these loci adds a further level of complexity and it has been shown to have a role in their instability. Many of the genes located within fragile sites are transcribed during late S phase leading to conflicts between the RNA pol II and replication [137]. This was demonstrated by the presence of R-loops at CFSs generated by clashing of a slower replication fork with the transcribing RNA pol II [58]. In this scenario, the RNA pol II, active during S phase, can displace assembled pre-recognition complexes at replication origins. Thus, late transcription of CFSs is another factor that prevents replication fork rescue by origin firing. In brief, dynamics of the different replication/transcription complexes within fragile sites affects stability of the loci. CFS are replicated and transcribed in a late stage of cell cycle and this may partially explain their instability. Fragile sites are replicated at late stages of S phase and are further delayed when a mild replication stress is present. This was initially demonstrated for FRA3B and then confirmed for many of the identified CFSs [132,138,139]. Although timing of replication affects stability of fragile sites, this is not the unique feature that makes them unstable, but rather the combination of their peculiar characteristics. Difficulties in replicating DNA during S phase may cause persistence of under replicated genomic loci at G2/early M phase [131,140,141]. The presence of under-replicated regions affects chromatin compaction during anaphase. The lower compaction of DNA can be visualized by the formation of ultra-fine anaphase bridges (UFBs) where under replicated DNA forms a physical link between the two homologues chromosomes that cannot be correctly segregated [142,143]. Mis-segregation of fragile sites determines their higher probability to generate breaks and gaps, especially after a mild replication stress. Gross chromosomal aberrations are not the sole consequence of genomic instability at the level of CFS. Copy number variations (CNVs) have also been identified as a consequence of the instability of these regions. When forks stall at fragile sites, a template switch mechanism is activated and finally intermediates are resolved through micro homology mediated repair leading to CNVs [144,145].

### 4.1. Replication of CFS

Studies of fragile sites have led to the identification of many of the characteristics that influence their replication. From the initial studies, it was already clear how efficient and stable replication is critical to prevent their instability. Notably, the initial identification of CFSs was made by observing chromosomal breaks following aphidicolin treatment [125]. In addition, defects in ATR, the main kinase involved in the replication stress response, cause breaks at CFS even in the absence of exogenous replication stress [146]. It is believed that the fork, while traveling through fragile sites, encounters a mild local replication stress. Such evidence further strengthens the correlation between a defective replication and CFSs instability. Stalling of the replication fork by local replication stress at fragile sites causes DNA entanglement between sister chromatids [147]. The entire Fanconi anemia pathway has been recognised as having an important role in preserving fragile sites stability. This is resolved by remodelling of the replication fork thanks to the activity of nucleases that assemble on the FA scaffold protein SLX4 together with FANCD2 [148]. In addition, regions bound by FANCD2 also presented unscheduled DNA synthesis at late G2/early M phase. FANCD2 seems to regulate also the activity of the BLM helicase that disentangles under-replicated DNA within anaphase or telophase bridges [149]. FANCD2 has been used as a bait to unravel CFSs interactors after aphidicolin treatment by mass spectrometry analysis, providing novel players in this pathway [150].

Recently, mitotic DNA synthesis was detected at fragile sites after oncogenic replication stress [143]. This synthesis is triggered by the MUS81-EME1 nucleases and depends on POLD3, a subunit of the polymerase δ replicative polymerase. Interestingly, RAD51 and BRCA2 cope with replication stress at CFSs during S phase but are dispensable during M phase DNA synthesis [151]. On the other hand, RAD52 is required and governs the assembly of the MUS81-EME1 complex and POLD3, precisely defining a spatial and temporal regulation of mitotic DNA synthesis. This system is part of a novel characterised DNA duplication pathway in mammalian cells, induced by DSBs. This has been named breaks induced replication (BIR) and is activated by the remodelling of collapsed forks mediated by nucleases [152,153]. Current models suggest a role of this DNA replication pathway as a last resort to complete duplication of fragile sites during mitosis. 

Given its pivotal role in providing a mechanism of DNA damage tolerance, translesion synthesis was predicted to have a role in replicating CFSs. Following local replication stress, alternative polymerases, in particular Y-family polymerases, may be recruited at the fork permitting fragile sites replication. Indeed, it was observed that depletion of polη in mammalian cells caused an increase of breaks at CFS even in the absence of replication stress [154]. Further studies showed the presence of polη at the level of FRA7H.1, FRA7H.2 and FRA16D by ChIP, thus strengthening the idea that this polymerase and a proficient translesion synthesis may be required to replicate past fragile sites [155]. The exchange between replicative and Y-family DNA polymerases at CFS has been reconstituted in vitro [156]. In particular, either polη or polκ were capable of elongating primers past CFS sequences substituting the pre-loaded PCNA-polδ complex. Altogether, this evidence strengthens the hypothesis of an involvement of translesion synthesis in preventing forks stress. However, a definitive demonstration of this activity in vivo is still elusive, and we lack a dynamic molecular model of the replication fork through these specific regions. 

### 4.2. CFS and Cancer

The presence, and instability, of fragile sites within coding sequences may have a direct impact on gene function. This is particularly relevant in the case of either tumour suppressors or oncogenes. The first and best characterized genes positioned in CFSs are FHIT and WWOX tumour suppressors [157,158,159]. Although the importance of FHIT as a genome caretaker has been clearly described, the role of the WWOX gene product is yet to be clearly defined [160]. Despite needing a better characterization of their role in genome instability at CSFs, both genes are clearly linked to cancer phenotypes in vivo. In accordance with this hypothesis, mice carrying deletions on either of the two tumour suppressors develop cancer with a higher frequency and show a higher sensitivity to treatments with carcinogenic agents [161,162]. Recent studies of cancer deletions, aimed to characterize large transcriptional units, have identified additional loci showing rearrangements, upon different replication stress [137]. This study demonstrated that breaks occurring in experimental conditions that affect DNA replication, such as aphidicolin, hydroxyurea and ionizing radiations, clustered at the same regions found in cancers. Indeed, CFS breaks are tissue-specific and cancers originating from different tissues show different CFS deletions, further confirming the tissue-specific patterns of CFSs expression [163,164]. Given their relevance for genomic instability in the presence of replication stress, studies on how these genomic regions are replicated may be of relevance in identifying novel therapeutic opportunities. The newly discovered correlation between FA and BIR in replication of fragile sites may become a source of promising druggable targets [165]. Some tumours show a RAD52 addiction, thus giving the opportunity to kill cancer cells by RAD52 inhibition. In line with this strategy, evidence has demonstrated synthetic lethality between RAD52 and other pathways involved in replication stress response. RAD52 showed synthetic lethality with both BRCA1 and BRCA2 [166,167]. Inhibitors of RAD52 have been designed, given its dispensable role in normal cells, however none of them have made it to the clinic yet [168,169,170,171,172].

## 5. Conclusions

Novel threats that lay within the genome have been characterized in the past few years. These have led to a completely new dynamic view on how the replication reacts to a series of previously overlooked obstacles. The discovery of R-loops has provided unprecedented insights into the interplay between genome replication and transcription, two of the main processes in all living organisms. Different studies have successfully characterized the consequences of R-loops on genome stability, suggesting that they present a clear obstacle to DNA replication. Despite this accurate characterization, we still lack an explanation on what is the physiological role of transient R-loops. On the basis of the present evidence, R-loops may have a role in controlling chromatin status. In line with this evidence, their presence might be linked to a higher chromatin compaction [23]. More intriguingly these hybrids map at either promoters or termination sites pointing to a role in gene expression regulation [14,20,23,27]. 

In future studies, the use of more sensitive methods to isolate and map R-loops may help to pinpoint not only stable hybrids but also transient structures. The majority of current methods rely on the use of the S9.6 antibody despite its broad specificity towards different nucleic acids containing RNA, such as RNA: RNA secondary structures. Although this approach has fostered new and exciting discoveries, it must be considered that the results obtained by DRIP could be somehow biased by specific structures that have higher affinity for the antibody. Furthermore, its use has required the establishment of robust control conditions. Despite these mitigations, the experimental evidence obtained by DRIP is not always consistent and has spurred debate in the field, especially regarding R-loop genome wide mapping [13]. These finding will need to be validated by alternative approaches with novel techniques that may take advantage of the affinity of RNase H for R-loops. In fact, by expressing a catalytically dead enzyme, it was possible to pull-down R-loops indirectly and map their position by sequencing [14,15]. Because RNase H has a higher specificity towards R-loops, such techniques will help isolating these structures in an unbiased in vivo context adding a fundamental piece of information on the physiological role of R-loops. Another big question regarding R-loops is their correlation with DNA damage. A direct causative connection between them is still hotly debated, and we still do not know if the accumulation of R-loops is sufficient to create damage or the opposite scenario could also be true, with DNA damage being the primary cause of R-loop formation. 

The characterization in vivo of G-quadruplex DNA has now opened new discussions on how the genome may be capable of auto-tuning gene expression by folding and melting its own regulatory sequences. At present, G4s have been detected in cells by using compounds that artificially stabilize their folding and bind indistinctively all across the genome. Although it has become clear that such structures are highly dynamic these approaches limit the possibilities of analysing changes of their folding in vivo. Moreover, whether an in vitro identified G4 structure may be stable or assume the same conformation in vivo remains elusive. Chemical synthesis of novel compounds to probe G4s in vivo will be fundamental in developing novel techniques that may definitively answer questions on G4 dynamic transitions. Another important issue to be addressed will be the specificity of ligands towards G4s. Even though subtle differences in terms of sequence binding can be identified among the different G4 ligands, they cannot target with precision a desired G4. This has led to pleiotropic effects that make difficult the interpretation of results obtained by G4 targeting. X-ray crystallography studies are now revealing differences between different G4s, raising the possibility to synthesize novel compounds directed towards a precise G4 target. Such compounds will help to study the role of a single G4 motif and may become essential to target with higher precision those motifs that have a clinical relevance. Finally, Common Fragile Sites are a potential source of damage because of their peculiar features. Their study is fundamental to unravel how the replication machinery handles difficult to replicate sequences, such as repeats. The evolutionary role of such sequences and in general of large transcribed loci is a debated topic in the field. The considerable length of these sequences is one of the sources of their instability. Thus, it remains unclear why these long sequences have been maintained instead of undergoing gene size reduction as frequently observed during evolution. Importantly, most of the length of these genes is given by very large introns compared to short exonic sequences. This may be a strategy evolved during evolution to buffer the fragility of these sequences and prevent loss of information. At present, we still lack definitive information on the evolutionary significance relevance of these loci. Interestingly, DNA breaks at very large genes have been identified in neuronal progenitors, where de novo CNVs may drive neurons development and differentiation but also predispose to neuronal pathologies and mental disorders [173].

A common thread between these genomic obstacles is their oncogenic potential. Failure to replicate these regions provokes replication stress, a hallmark of cancer cells. Remarkably, players involved in their metabolism are on the spotlight as new candidates of chemotherapeutics that may hijack these networks to kill cancer cells. Considering the recent characterization of some of the players in these pathways, many questions remain unanswered. Nevertheless, it is clear that a more detailed analysis of the function of these DNA structures will help increasing our understanding of DNA replication and may be rewarded by the identification of potential novel targets to be exploited in the clinic.

## Abbreviations

ATR	Ataxia Telangectasia and Rad3 Related
FANCM	Fanconi Anemia Complementation Group M
FANCD2	Fanconi Anemia Complementation Group D2
FANCI	Fanconi Anemia Complementation Group I
SLX4	SLX4 Structure-Specific Endonuclease Subunit
MUS81	gene name
XPF	Xeroderma Pigmentosum Group F
Rad6	Radiation gene 6 homolog
SMARCAL1	SWI/SNF related, matrix associated, actin dependent regulator of chromatin, subfamily a like 1
ZRANB3	zinc finger RANBP2-type containing 3
HLTF	helicase like transcription factor
SHPRH	SNF2 histone linker PHD RING helicase
BRCA1	BReast CAncer 1
BRCA2	BReast CAncer 2
WRN	Werner syndrome RecQ like helicase
RECQ1	RecQ Protein-Like 1
ChIP	Chromatin ImmunoPrecipitation
AQR	aquarius intron-binding spliceosomal factor
DDX19	DEAD-box helicase 19
DDX23	DEAD-box helicase 23
DDX1	DEAD-box helicase 1
DDX5	DEAD-box helicase 5
DHX9	DExH-box helicase 9
BLM	Bloom syndrome RecQ like helicase
XPG	Xeroderma Pigmentosum Group F
PCID2	PCI domain containing 2
TREX-2	Transcription and export complex 2
Mre11	meiotic recombination 11 homolog
EME1	essential meiotic structure-specific endonuclease 1
Chk1	checkpoint kinase 1
HRAS	Harvey rat sarcoma viral oncogene homolog
EWS-FLI1	EWS RNA binding protein 1- Friend leukemia virus integration 1 fusion gene
PARP	poly(ADP-ribose) polymerases
Pif1	PIF1 5′-to-3′ DNA helicase
Rev1	REV1 DNA directed polymerase
DT40	chicken B cell line derived from an avian leukosis virus (ALV)-induced bursal lymphoma
BU-1	Chicken B-cell marker chB6
DNMT1	DNA methyltransferase 1
cMYC	MYC proto-oncogene, bHLH transcription factor
KRAS	Kirsten rat sarcoma viral oncogene homolog
c-KIT	v-kit Hardy-Zuckerman 4 feline sarcoma viral oncogene homolog
BCL2	BCL2 apoptosis regulator
VEGF	vascular endothelial growth factor A
RHSP4	Telomerase inhibitor
EMICORON	G4-interactive molecule
HCT116 Human	COLORECTAL CARCINOMA cell line
HEK293T	human embryonic kidney 293 T
FHIT	ragile histidine triad diadenosine triphosphatase
WWOX	WW domain containing oxidoreductase
POLD3	DNA polymerase delta 3
